# Quality over quantity: association between autistic traits and daily-life social interactions in adolescents and young adults from the TwinssCan cohort

**DOI:** 10.1017/S1092852926100947

**Published:** 2026-06-01

**Authors:** Laura Fusar-Poli, Thanavadee Prachason, Natascia Brondino, Marta Nola, Bochao D. Lin, Lotta-Katrin Pries, Melike Karaçam Doğan, Claudia Menne-Lothmann, Jeroen Decoster, Ruud van Winkel, Dina Collip, Philippe Delespaul, Marc De Hert, Catherine Derom, Evert Thiery, Nele Jacobs, Jim van Os, Bart PF Rutten, Pierluigi Politi, Sinan Guloksuz

**Affiliations:** 1 Department of Brain and Behavioral Sciences, University of Pavia, Pavia, Italy; 2 Istituti Clinici Scientifici Maugeri IRCCS, Pavia, Italy; 3 Department of Psychiatry, Faculty of Medicine, Ramathibodi Hospital, Mahidol University, Bangkok, Thailand; 4 Department of Psychiatry and Neuropsychology, Mental Health and Neuroscience Research Institute, Maastricht University Medical Centre, Maastricht, Netherlands; 5 Department of Psychiatry, Ankara Bilkent City Hospital, Ankara, Türkiye; 6 University Psychiatric Centre, KU Leuven, Leuven, Belgium; 7 Antwerp Health Law and Ethics Chair, AHLEC University Antwerpen, Antwerp, Belgium; 8 Centre of Human Genetics, University Hospitals Leuven, Leuven, Belgium; 9 Department of Obstetrics and Gynaecology, Ghent University Hospitals, Ghent, Belgium; 10 Department of Neurology, Ghent University Hospitals, Ghent, Belgium; 11 Faculty of Psychology, Open University of the Netherlands, Netherlands; 12 Division Neuroscience, University Medical Centre Utrecht, Utrecht, Netherlands; 13 Department of Psychosis Studies, Institute of Psychiatry, King’s Health Partners, King’s College London, London, UK; 14 Department of Psychiatry, University of British Columbia, Vancouver, Canada; 15 Institute of Mental Health, University of British Columbia, Vancouver, Canada; 16 Northern Medical Program, University of Northern British Columbia, Prince George, Canada; 17 Department of Psychiatry, Yale School of Medicine, New Haven, United States

**Keywords:** Autistic traits, social interaction, daily life, ecological momentary assessment, psychopathology

## Abstract

**Objective:**

Autistic traits (ATs) are associated with difficulties in social functioning, but their impact on the quantity and the quality of daily-life social interactions is not yet fully understood. Hereby, we examined the relationship between AT and daily-life social interactions in adolescents and young adults.

**Methods:**

Data were derived from the TwinssCan cohort (*N* = 593). ATs were assessed using the Autism Spectrum Quotient (AQ), while the quantity and the quality of daily-life social interactions were evaluated using the Experience Sampling Methodology. Multilevel regressions were performed, with the AQ as the independent variable and each social/solitary quantity/quality variable as the dependent variable. Models were adjusted for age, sex, and general psychopathology, measured with the Symptom Checklist-90.

**Results:**

No significant association was found between AQ total scores and quantity of social interactions. However, AQ total scores were significantly associated with quality of social interactions. Higher ATs were associated with an increased preference to be alone (*B* = 0.04, 95% CI 0.01–0.07), less pleasure while in-company (*B* = −0.02, 95% CI −0.04 to −0.01), less feeling of safety while in-company (*B* = −0.02, 95% CI −0.04 to −0.002), and less feeling of belongingness (*B* = −0.02, 95% CI −0.03 to −0.001). ATs were differently associated with the quality of social interactions depending on the familiarity. No significant associations were found between ATs and solitary qualities.

**Conclusions:**

Our findings highlight the importance of evaluating ATs during clinical assessments, especially in adolescents and young adults, to evaluate the impact on social interactions and the potential psychopathological consequences.

## Introduction

Autism spectrum disorder (ASD) is a neurodevelopmental condition characterized by persistent deficits in social communication and social interaction across multiple contexts.^1^ In ASD, altered social interactions emerge very early on and remain relatively stable or slightly improve with age,^2^^,^^3^ inducing difficulties in social reciprocity as well as in initiating and maintaining social interactions and relationships.^4^ These differences in social functioning and social skills can lead to lower social participation and higher isolation that are associated with broad negative consequences and poor life-related long-term outcomes.^2^^,^^5^

There is evidence that autistic traits (ATs) are distributed along a continuum in the general population^6^^,^^7^ and remain stable along the lifespan.^8^ ATs are negatively associated with self-reported social functioning in neurotypical individuals.^9^ Moreover, elevated ATs seem to be associated with a higher vulnerability to psychopathology.^10^^–^^17^

Social interactions are fundamental for daily life. In patient populations, both the quantity of social behavior (eg, the amount of time spent with others) and its experiential quality (eg, how comfortable one is when with others) have been associated with psychopathology. For instance, fewer social interactions and more negatively experienced social interactions have been reported for people with depressive, anxiety, and psychotic disorders.^18^^–^^20^

Several methods can be used to assess social processes as they manifest in daily life.^21^ Self-reported questionnaires and clinical interviews with study participants or their relatives may present several limitations, such as social desirability biases and recall biases.^22^ Additionally, such approaches are specifically and exclusively focused on the frequency of social activities. However, social interactions can occur across a wide range of contexts, including brief talks in the workplace or at the supermarket. Thus, the previous approach may not be sufficiently reliable for comprehensively evaluating the totality of daily social behaviors.^23^ Additionally, to fully understand social interactions in daily-life contexts, it is important to focus not only on the quantity of social behavior (eg, the amount of time spent with others) but also on its experiential quality (eg, how comfortable one is when with others). As highlighted by recent research, the distinction between objective and subjective social experiences is relevant to consider, since subjective aspects of social interactions do not necessarily relate to objective aspects; additionally, subjective aspects might be more strongly related to outcomes, particularly in young people.^24^

In this context, the Experience Sampling Method (ESM) may represent a unique opportunity to assess social processes, as it allows for more ecologically valid assessments of social interactions in daily life, focusing on both the objective and subjective experience. The ESM is a research technique where participants report on their real-time feelings, thoughts, and behaviors multiple times a day in their natural environment, often via smartphone prompts, to capture momentary experiences accurately, avoiding retrospective bias and providing ecological validity for understanding daily life patterns.^25^^,^^26^

Previous ESM studies have assessed social interactions of people with a clinical diagnosis of ASD.^23^^,^^27^^,^^28^ However, to the best of our knowledge, no research has evaluated the association between ATs and daily-life social interaction in a general population sample. In the framework of increasing attention to autism-related conditions, a growing number of studies have recently investigated the prevalence and features of ATs in non-clinical populations. Therefore, to better investigate the impact of ATs on daily life social interactions in non-autistic people, the present study aimed to (1) examine the association between ATs and the quantity and quality of daily-life social interactions in adolescents and young adults using ESM and (2) examine the interacting effects of being with familiar or non-familiar company and ATs on the quality of social interaction.

## Methods

### Participants

Adolescent and adult twins aged 15–35 and their non-twin siblings (*N* = 778) were recruited as part of the TwinssCan study^29^ sampled from the population-based multiple-birth registry East Flanders Prospective Twin Survey in the Dutch-speaking Flanders region in Belgium.^30^ The TwinssCan study included a battery of questionnaires and experimental tasks, in addition to ESM data collection. Data from 667 twins and siblings with available ESM data were included in the present study. Exclusion criteria included lack of parental or caregiver consent for participants under 18, and the presence of a severe and chronic psychiatric disorder, as reported by caregivers, consistent with the Dutch consensus definition of severe mental illness,^31^ such as schizophrenia, other psychotic disorders, bipolar disorder with psychotic features, ASD, or substance use disorders, when these conditions substantially impaired functioning and required coordinated care. Conforming to previous studies,^32^^,^^33^ we excluded those who completed less than one-third of the ESM questionnaires (*n* = 57). Additional 17 participants were excluded due to missingness of the predicting variables, leaving data from 593 participants (180 monozygotic twins, 378 dizygotic twins, and 35 siblings) for final analyses. Written informed consent was obtained from all participants and from their parents, if subjects were younger than 18. The study was approved by the local ethics committee (Ethics Committee Research UZ/KU Leuven, Nr. B32220107766).

### Measures

#### Autistic traits

The Autism Spectrum Quotient (AQ-50) is a self-report tool that measures the degree to which an individual aged 16 or older with average or above-average intelligence has the traits associated with the autism spectrum.^34^ It includes 50 items with a 4-point Likert scale ranging from “Totally agree” to “Totally disagree.” The Dutch translation of the AQ was used for this study.^35^ According to the most widely used scoring method, a score of 0 or 1 can be attributed to each item and the total score can thus range between 0 and 50. The AQ consists of five subscales measuring the following domains: Social skill, Attention switching, Attention to detail, Communication, and Imagination. Higher scores on both the AQ total and subscales are suggestive of higher ATs.

#### General psychopathology

The Dutch version of the Symptom Checklist-90 (SCL-90) is a self-report questionnaire consisting of 9 subscales and 90 items with Likert scales ranging from 0 (“not at all”) to 4 (“extremely”).^36^ The SCL-90 was used to construct a global psychopathology score, the Global Severity Index (GSI), which represents the mean across all SCL-90 items.^37^

### Experience sampling method (ESM)

ESM is a research technique where participants report on their real-time feelings, thoughts, and behaviors multiple times a day in their natural environment. In the present study, participants answered ESM questionnaires on the PsyMate™, a custom-made Personal Digital Assistant.^38^ In the six-day ESM period, participants received 10 prompts per day, between 7.30 AM and 10.30 PM, and each prompt contained a maximum of 57 items (subject to conditional branching). Participants had 15 minutes to respond to the questionnaire following each prompt. Participants were instructed to answer items asking who they were with and how they felt just at the moment before the prompt. Details of the prompts are presented in Supplementary Figure S1.

#### Quantity of social interactions

Participants were asked at each prompt who they were with, and they could select one or multiple of different types of company. Conforming to a prior study,^24^ three binary context variables were generated for each momentary assessment: (1) being alone (1 = alone, 0 = with company); (2) being with familiar people, including partners, relatives, and friends (1 = with familiar people, 0 = without familiar people); and (3) being with less-familiar people, including colleagues, acquaintances, and strangers/others (1 = with less-familiar people, 0 = without less-familiar people). To ensure distinctive social situations, three mutually exclusive social contexts (ie, with familiar people only, with less-familiar people only, and mixed) were generated and used in a sensitivity analysis.

#### Quality of social interactions and solitude

When participants were in-company, they were asked to rate the degree to which they preferred being alone, felt safe in-company, found company pleasant, felt judged, and felt belonged in the moment. When participants were alone, they were asked to rate the degree to which they found being alone pleasant, felt safe being alone, and preferred being in-company. All solitary and social quality items were rated on a scale from 1 (“Not”) to 7 (“Very”). The higher the score, the more they agreed with such experiences.

### Statistical analysis

All statistical analyses were conducted using Stata software version 16.0.^39^ Stata code used for analysis is available online on the OSF-page for this project (https://osf.io/pnxsg). Multilevel regression analyses with random intercepts were used to account for the hierarchical structure of the dataset. Multiple ESM observations (level 1) were clustered within subjects (level 2), which were nested within families (level 3). We conducted the main analyses using the total AQ score as the independent variable and secondary exploratory analyses using the AQ subscale scores as the independent variables.

To test the associations between ATs and the quantity of social interactions/solitude, multilevel logistic regression was performed, using each binary social context variable as the dependent variable. For the quality of social interactions or solitude, we first visualized the distributions of each outcome variable and selected multilevel tobit regression for the outcomes that showed ceiling (ie, find company pleasant, feel safe in-company, feel belonged, and feel safe when alone) or floor effects (ie, prefer to be alone and feel judged) and multilevel linear regression for the outcomes that did not significantly deviate from normality (ie, pleasant when alone and prefer company). Standardized regression coefficients based on the total variance (the sum of all random-effect and residual variance components) were also reported to facilitate comparison across different outcome measures. To investigate whether different social situations have differential effects on social quality among people with different ATs levels, an interaction term between the presence of familiar or less-familiar people (no = 0, yes = 1) and ATs on social quality was added to the models.

Age, sex, and general psychopathology measured with the GSI score of the SCL-90 were included as covariates in each model. Statistical significance for the main analyses was set at p < 0.05. To maximize the power of analyses and given the explorative nature of our study, no multiple testing correction was applied for primary analyses to avoid an increase in Type II error.^40^ For secondary exploratory analyses using the AQ subscale scores, Bonferroni correction for multiple testing was applied (Bonferroni-corrected *p* < 0.01), and *q*-values controlling for 5% false discovery rate were derived by the Benjamini–Yekutieli procedure.^41^

To ensure robustness of the findings, sensitivity analyses were performed: (1) using multilevel mixed-effects ordered logistic regression instead of multilevel tobit or linear regression, (2) additionally adjusting for temporal fluctuations by including a binary indicator for day type (weekend vs. weekday) and modeling time-of-day via linear marginal splines (with knots at 12:00 and 17:00), alongside the total number of beep responses; and (3) using three mutually exclusive social contexts (ie, with familiar people only, with less-familiar people only, and mixed) as an alternative independent variable in the interaction analysis.

## Results

### Characteristics of participants

Descriptive characteristics of the sample and compliance with ESM prompts were reported in Table 1.

**Table 1.** Characteristics of Participants (*N* = 593) Table 1. long description.

	Mean (SD)	Median (IQR)
**Age (years)**	17.6 (3.8)	16.0 (15.0–18.0)
**Female, n (%)**	366 (61.7)	
**Total AQ**	15.6 (5.8)	15.0 (12.0–19.0)
**Social skill**	2.1 (2.0)	2.0 (1.0–3.0)
**Attention switching**	3.8 (1.9)	4.0 (2.0–5.0)
**Attention to detail**	4.4 (2.1)	4.0 (3.0–6.0)
**Communication**	2.5 (1.8)	2.0 (1.0–4.0)
**Imagination**	2.8 (1.8)	3.0 (1.0–4.0)
**Total SCL-90 (GSI score)**	0.5 (0.4)	0.4 (0.2–0.6)
**Ecological momentary assessment**		
**Number of completed beeps**	39.6 (9.1)	39 (33–46)
**Social quantities**		
**% Time with any company**	78.6 (16.8)	81.1 (70.0–92.1)
**% Time with familiar people^a^**	91.8 (14.7)	97.7 (91.9–100)
**% Time with less-familiar people^a^**	26.2 (20.3)	23.4 (9.5–40.0)
**Social qualities**		
**Prefer to be alone**	1.8 (1.4)	1.0 (1.0–2.0)
**Find company pleasant**	5.7 (1.3)	6.0 (5.0–7.0)
**Feel safe in-company**	5.9 (1.3)	6.0 (5.0–7.0)
**Feel judged**	2.3 (1.6)	2.0 (1.0–3.0)
**Feel belonged**	5.9 (1.2)	6.0 (5.0–7.0)
**Solitary qualities**		
**Pleasant when alone**	4.6 (1.8)	5.0 (3.0–6.0)
**Prefer company**	3.7 (1.8)	4.0 (2.0–5.0)
**Feel safe when alone**	5.7 (1.3)	6.0 (5.0–7.0)

*Note:* AQ, Autism-Spectrum Quotient; GSI, Global Severity Index; IQR, interquartile range; SCL-90, Symptom Checklist-90 Revised; SD, standard deviation.

a The percentage was calculated based on the overall time being in company.

### Association between autistic traits and quantities and quality of social interactions

As reported in Table 2, no significant associations were found between ATs and the quantity of social interactions. We found significant associations between ATs and social qualities. Particularly, higher ATs were associated with increased preference to be alone (*B* = 0.04, 95% CI 0.01 to 0.07, *p* = 0.01), less pleasure while in-company (*B* = −0.02, 95% CI −0.04 to −0.01, *p* = 0.008), less feeling of safety while in-company (*B* = −0.02, 95% CI −0.04 to −0.002, *p* = 0.03), and less feeling of belongingness (*B* = −0.02, 95% CI −0.03 to −0.001, *p* = 0.04). No significant association was found between ATs and solitary qualities. Sensitivity analyses using multilevel mixed-effects ordered logistic regression or additionally accounting for time of day, day type, and the total number of beep responses yielded comparable findings (Supplementary Tables S1–2).

**Table 2.** Associations of Autistic Traits with the Quantities of Social Interactions and the Qualities of Social or Solitary Experiences Table 2. long description.

Social quantities	B (95% CI)	OR (95% CI)	p
With any company	0.01 (−0.01 to 0.02)	1.01 (0.99 to 1.02)	0.48
With familiar people	−0.01 (−0.04 to 0.02)	0.99 (0.96 to 1.02)	0.60
With less-familiar persons	−0.003 (−0.02 to 0.02)	1.00 (0.98 to 1.02)	0.74

Social qualities	B (95% CI)	β (95% CI)	p
Prefer to be alone	0.04 (0.01 to 0.07)	0.05 (0.01 to 0.09)	**0.01**
Find company pleasant	−0.02 (−0.04 to −0.01)	−0.06 (−0.11 to −0.02)	**0.008**
Feel safe in-company	−0.02 (−0.04 to −0.002)	−0.05 (−0.10 to −0.01)	**0.03**
Feel judged	0.01 (−0.01 to 0.04)	0.02 (−0.04 to 0.07)	0.35
Feel belonged	−0.02 (−0.03 to −0.001)	−0.06 (−0.10 to −0.01)	**0.04**

Solitary qualities	B (95% CI)	β (95% CI)	p
Pleasant when alone	0.01 (−0.02 to 0.03)	0.02 (−0.05 to 0.09)	0.61
Prefer company	−0.01 (−0.03 to 0.01)	−0.04 (−0.11 to 0.03)	0.25
Feel safe when alone	−0.01 (−0.04 to 0.01)	−0.04 (−0.11 to 0.02)	0.29

*Note:* All models were adjusted for age, sex, and total SCL-90. Statistical significance (*p* < 0.05) is presented in bold. Standardized coefficients were calculated based on the total latent variance (the sum of all random-effect and residual variance components). AQ, Autism Spectrum Quotient; B, unstandardized regression coefficient; CI, confidence interval; OR, odds ratio; β, standardized regression coefficient.

Secondary exploratory analyses conducted using the AQ subscales as independent variables confirmed a significant positive association between the AQ Social Skill subscale and a preference to be alone when in-company (*B* = 0.15, 95% CI 0.06–0.23, *p* < 0.001, *q* = 0.01), indicating that the more social skill deficits, the higher the preference to be alone when in-company. Additionally, a negative association between the AQ Social Skill subscale and the pleasure of company (*B* = −0.08, 95% CI −0.12 to −0.03, *p* < 0.001, *q* = 0.009) was found, indicating that the more social skill deficits, the lower the pleasure of being in-company. The results of secondary analyses were reported in Supplementary Table S3. Multilevel mixed-effects ordered logistic regression also yielded similar findings (Supplementary Table S4).

### Interacting effects of autistic traits and the presence of familiar or less-familiar people on social qualities

**Figure 1.**
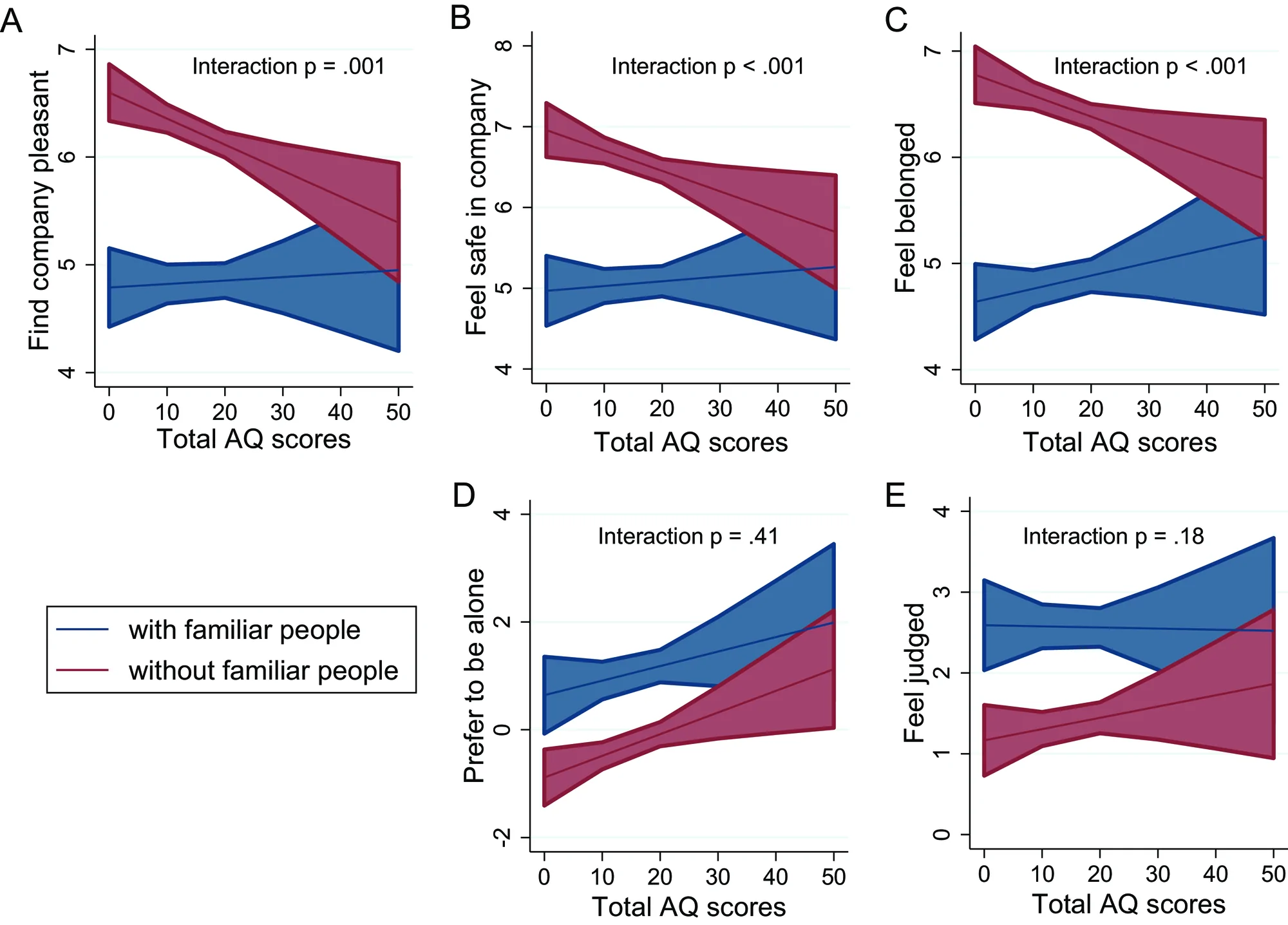
Marginal predictions of ratings for the quality of social interactions in the presence or absence of familiar people, varying by AQ scores. Predicted means with 95% CI and p-values of the interaction between total AQ and social context are presented. (A) Finding company pleasant, (B) feeling safe in company, (C) feeling belonged, (D) prefer to be alone, and (E) feeling judged. Figure 1. long description.

**Figure 2.**
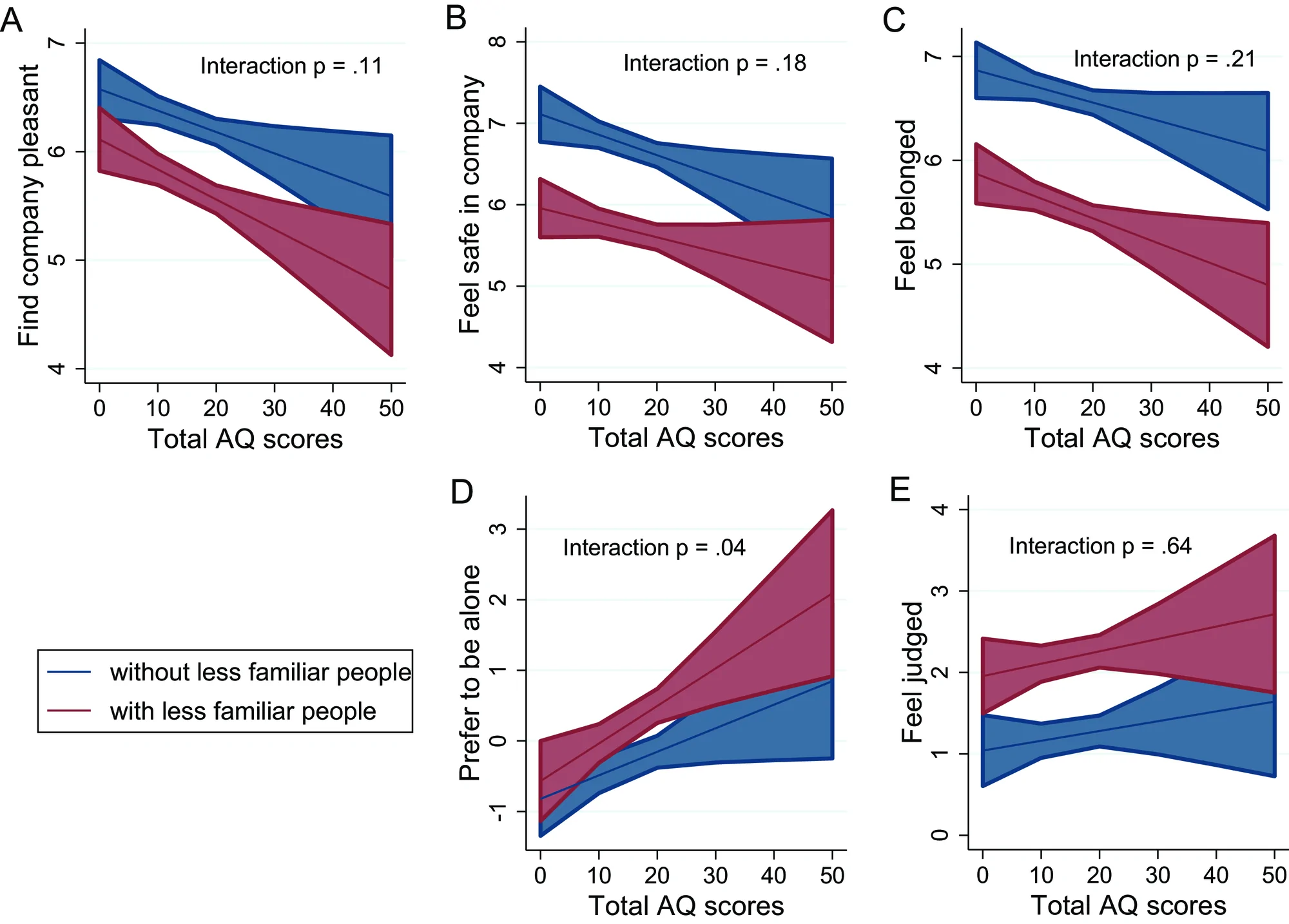
Marginal predictions of ratings for the quality of social interactions in the presence or absence of less-familiar people, varying by AQ scores. Predicted means with 95% CI and p-values of the interaction between total AQ and social context are presented. (A) Finding company pleasant, (B) feeling safe in company, (C) feeling belonged, (D) prefer to be alone, and (E) feeling judged. Figure 2. long description.

As reported in Table 3, the association between ATs varied according to the familiarity of the company. Particularly, when staying with familiar people, ATs were associated with decreased pleasure in-company (*B* = −0.03, 95% CI −0.04 to −0.01, *p* = 0.001), less feeling of safety in-company (*B* = −0.03, 95% CI −0.05 to −0.01, *p* < 0.001), and decreased sense of belonging (*B* = −0.03, 95% CI −0.05 to −0.02, *p* < 0.001). Marginal linear prediction showed that, in the presence of familiar people, a 10-point increase in AQ score predicted a 0.24-point decrease in feeling pleasant, a 0.25-point decrease in feeling safe, and a 0.20-point decrease in feeling belonged (Figure 1A–C). When staying with less-familiar people, ATs were only positively associated with a preference to be alone (*B* = 0.02, 95% CI 0.001 to 0.04, *p* = 0.04). Marginal linear prediction showed that, in the presence of less-familiar people, a 10-point increase in AQ score predicted a 0.53-point increase in preference to be alone (Figure 2D). The sensitivity analysis using three mutually exclusive categories of social context revealed similar findings, showing that ATs were associated with finding company less pleasant, feeling less safe, and feeling less belonged in the presence of familiar people, regardless of the presence of less-familiar people (Supplementary Figure S2A-C). On the other hand, in the presence of less-familiar people, high ATs were associated with a preference to be alone despite the presence of familiar people (Supplementary Figure S2D). The sensitivity analyses using multilevel mixed-effects ordered logistic regression or additionally accounting for time of day, day type, and the total number of beep responses also confirmed the significant findings (Supplementary Tables S5–6).

**Table 3.** Interacting Effects of Autistic Traits and the Presence of Familiar or Less-Familiar People on Social Qualities Table 3. long description.

Social qualities	With familiar people		With less-familiar people	
	B (95% CI)	p	B (95% CI)	p
Prefer to be alone	0.01 (−0.02 to 0.04)	0.41	0.02 (0.001 to 0.04)	**0.04**
Find company pleasant	−0.03 (−0.04 to −0.01)	**0.001**	−0.01 (−0.02 to 0.002)	0.11
Feel safe in-company	−0.03 (−0.05 to −0.01)	**<0.001**	0.01 (−0.003 to 0.02)	0.18
Feel judged	0.02 (−0.01 to 0.04)	0.18	0.003 (−0.01 to 0.02)	0.64
Feel belonged	−0.03 (−0.05 to −0.02)	**<0.001**	−0.01 (−0.01 to 0.003)	0.21

*Note:* All models were adjusted for age, sex, and total SCL-90. Statistical significance (*p* < 0.05) is presented in bold. AQ, Autism Spectrum Quotient; B, unstandardized regression coefficient; CI, confidence interval.

As reported in Supplementary Table S7, results were confirmed in secondary analyses conducted using the AQ Social Skill subscale. When staying with familiar people, more social skills deficits were associated with decreased pleasure in-company (*B* = −0.08, 95% CI −0.13 to −0.03, *p* = 0.002, *q* = 0.002), less feeling of safety in-company (*B* = −0.09, 95% CI −0.14 to −0.03, *p* = 0.003, *q* = 0.011), and decreased sense of belonging (*B* = −0.08, 95% CI −0.03 to −0.04, *p* < 0.001, *q* = 0.003). Linear predictions of social qualities in the presence or absence of familiar and non-familiar people varying by the AQ Social Skill subscale are presented in Supplementary Materials (Figure S3 and Figure S4, respectively). The sensitivity analysis using multilevel mixed-effects ordered logistic regression yielded comparable results (Supplementary Table S7).

## Discussion

The present study explored, for the first time, the association between ATs and the quantity and quality of daily-life social interactions measured using the ESM approach in a large cohort of 593 adolescents and young adults from the general population. Although our results did not show any significant interaction between ATs and the quantity of social interactions, we found significant associations between ATs and the momentary quality of social interactions. Overall, our findings suggest that quality over quantity matters.

The non-significant association between ATs and quantity of social interactions is in line with previous studies adopting the ESM approach, which reported no differences in the frequency of social interaction in an adult autistic population^23^ and pervasive developmental disorders^42^ in comparison with neurotypical controls.

We found significant associations between ATs and the quality of social interactions, indicating that ATs may influence the subjective experience of social interactions. Specifically, while in-company, ATs were positively associated with an increased preference to be alone, less pleasure, less feeling of safety, and less feeling of belongingness. Such associations were not confirmed in the case of solitary qualities, further supporting the specificity of these findings for social interactions. This finding is in line with a recent ESM study in which autistic participants reported feeling more excluded, experimenting with worse social experiences, and desiring to be alone when in-company, compared with neurotypical individuals.^43^ Interestingly, no association was found between ATs and the feeling of being judged. From a clinical point of view, this observation may be useful to discriminate between ATs and avoidant personality traits, which are typically associated with concern about criticism or disapproval.^1^ Of note, the participants included in the present study acknowledged, on average, low levels of ATs, which may have an impact on the quality of social interactions, without significantly affecting the quantity of those interactions.

Main results were confirmed in secondary analyses conducted on the AQ Social Skill subscale. Conversely, other AQ subscales were not significantly associated with the quality of social interactions, suggesting a prominent role of social skills in subjective social experiences. Our findings might be explained by the intrinsic difficulties in social communication experienced by autistic individuals or individuals with elevated ATs. Nevertheless, it is also important to consider that such impairments are not unidirectional. The mismatch in communication and interaction styles between non-autistic and autistic people or individuals with elevated ATs (eg, Crompton et al.^44^) can contribute to atypical social exchanges, thus worsening the quality of the interaction.

Additionally, it is worth mentioning that the AQ does not include the whole range of the autism spectrum. For instance, restricted interests and repetitive behaviors, which may also be relevant for the quality of interactions,^45^ are not evaluated in the AQ. Similarly, atypical sensory profiles may be implicated in a wide array of autistic social difficulties. Social interactions can indeed manifest as sensorially disturbing, chaotic, and unpredictable,^46^ thus negatively impacting the subjective experience of individuals with elevated ATs. Another reason could be related to the higher levels of paranoid-like ideation in people with elevated ATs, given both difficulties in understanding others’ mental states and frequent experiences of negative social interactions.^47^ Also, anxiety levels could have an impact, as evidenced by a recent meta-analysis showing a negative, but small, impact of anxiety on social competence measures in the autistic population. Young people with elevated ATs may become anxious about being perceived as having poorer social competencies due to ongoing feelings of being misunderstood or rejected. Indeed, autistic people describe experiences of acceptance and belongingness as crucial aspects of their well-being.^48^ However, previous research reported that autistic individuals would not prefer to be alone when in-company, even when experiencing anxiety,^42^^,^^49^ partially in contrast with this hypothesis.

Our results showed that the quality of social interactions varied in the presence of familiar and less-familiar people. In fact, a significant positive association between ATs and a preference for being alone was retrieved only in the case of interactions with less-familiar people. Conversely, in the case of interaction with familiar people, a 10-point increase in AQ score predicted a 0.24-point decrease in feeling pleasant, a 0.25-point decrease in feeling safe, and a 0.20-point decrease in feeling belonged. This finding is partially in contrast with previous research on the autistic population. For instance, Hintzen and colleagues^42^ reported that people with ATs did not seem to evaluate the social context, in contrast with neurotypical individuals. In both groups, increasing the level of non-familiarity was associated with an increased preference for being alone and a reduction in pleasure while in-company. Although our results might seem counterintuitive, we could speculate that, when in the company of familiar people, individuals with higher ATs may perceive social obligations that feel more demanding or stressful (eg, needing to behave in a certain way). People with elevated ATs may also try harder to “mask” or hide difficulties when with familiar people, which can be exhausting and reduce enjoyment. Because familiar social contexts are typically associated with safety and comfort, negative experiences in these settings may be felt more intensely when expectations are not met. Also, the feeling of being judged might be increased in the presence of familiar company. Conversely, when interacting with non-familiar individuals, the implicit expectation to engage in “small talk” may be perceived as less relevant, and any difficulties in interaction may feel less important to conceal. These observations are speculative in nature, and additional research is necessary to confirm the proposed hypotheses.

The results of the present study should be interpreted in light of some limitations. First, we focused on ATs in the general population and not on a clinical sample, thus hampering the generalizability of our findings to the whole autism spectrum. However, it is well-established that the autism spectrum has a wide range of subclinical expressions, which may be equally relevant in the development of psychopathological conditions. Second, we focused on a population of adolescents and young adults, which is the age group in which mental disorders typically emerge.^50^^,^^51^ As the sample predominantly comprises younger individuals, the results may not be generalizable to children or adult populations. However, it has been shown that ATs are stable throughout the lifespan.^8^ Third, potential confounding variables such as intelligence quotient, socioeconomic status, and attention deficit-hyperactivity symptoms were not included in regression analyses. Also, alexithymia—characterized by difficulty identifying and describing one’s own emotions—may contribute to impaired recognition of others’ emotions and poor emotional regulation.^52^ This can, in turn, increase social anxiety, reduce social engagement, and exacerbate difficulties in social interaction.^53^^,^^54^ Finally, the ESM is a time-consuming measure, which requires participants to respond to several prompts during the day. Therefore, participants’ compliance might have been affected by the time and commitment required. However, excluding participants who have responded to less than 30% of the questionnaires makes our results undoubtedly more accurate and reliable.

## Conclusion

The present study suggests that ATs are associated with the quality, rather than the quantity, of social interactions in adolescents and young adults from the general population. Our findings highlight the need to investigate the presence and relevance of ATs during clinical assessments. Clinicians should focus not exclusively on the quantity of relationships and interactions (eg, number of friendships, number of out-of-home activities) but rather on the quality and subjective experience of social interactions. Exploring individual perceptions of social connections may help in recognizing the mechanisms underlying difficulties in social communication. Eventually, working on the associated negative mechanisms may be useful to prevent the development of co-occurring psychopathological conditions arising from poor or atypical social interactions.

## Supporting information

10.1017/S1092852926100947.sm001 Fusar-Poli et al. supplementary material Fusar-Poli et al. supplementary material

## Supplementary material

The supplementary material for this article can be found at http://doi.org/10.1017/S1092852926100947.

## Table 1. Long description

The table contains three columns: variable, mean with standard deviation, and median with interquartile range. The first section lists participant characteristics. Age has a mean of 17.6 with standard deviation 3.8, median 16.0 with I Q R 15.0 to 18.0. Female count is 366, 61.7 percent. Total A Q mean is 15.6, standard deviation 5.8, median 15.0, I Q R 12.0 to 19.0. Social skill mean is 2.1, standard deviation 2.0, median 2.0, I Q R 1.0 to 3.0. Attention switching mean is 3.8, standard deviation 1.9, median 4.0, I Q R 2.0 to 5.0. Attention to detail mean is 4.4, standard deviation 2.1, median 4.0, I Q R 3.0 to 6.0. Communication mean is 2.5, standard deviation 1.8, median 2.0, I Q R 1.0 to 4.0. Imagination mean is 2.8, standard deviation 1.8, median 3.0, I Q R 1.0 to 4.0. Total S C L dash 90 G S I score mean is 0.5, standard deviation 0.4, median 0.4, I Q R 0.2 to 0.6. The next section, ecological momentary assessment, lists number of completed beeps mean 39.6, standard deviation 9.1, median 39, I Q R 33 to 46. Social quantities include percent time with any company mean 78.6, standard deviation 16.8, median 81.1, I Q R 70.0 to 92.1; percent time with familiar people mean 91.8, standard deviation 14.7, median 97.7, I Q R 91.9 to 100; percent time with less-familiar people mean 26.2, standard deviation 20.3, median 23.4, I Q R 9.5 to 40.0. Social qualities include prefer to be alone mean 1.8, standard deviation 1.4, median 1.0, I Q R 1.0 to 2.0; find company pleasant mean 5.7, standard deviation 1.3, median 6.0, I Q R 5.0 to 7.0; feel safe in-company mean 5.9, standard deviation 1.3, median 6.0, I Q R 5.0 to 7.0; feel judged mean 2.3, standard deviation 1.6, median 2.0, I Q R 1.0 to 3.0; feel belonged mean 5.9, standard deviation 1.2, median 6.0, I Q R 5.0 to 7.0. Solitary qualities include pleasant when alone mean 4.6, standard deviation 1.8, median 5.0, I Q R 3.0 to 6.0; prefer company mean 3.7, standard deviation 1.8, median 4.0, I Q R 2.0 to 5.0; feel safe when alone mean 5.7, standard deviation 1.3, median 6.0, I Q R 5.0 to 7.0. Section and variable labels are bolded. Footnotes clarify that percentages for familiar and less-familiar people are based on overall time in company. Abbreviations: A Q is Autism-Spectrum Quotient, G S I is Global Severity Index, I Q R is interquartile range, S C L dash 90 is Symptom Checklist 90 Revised, S D is standard deviation.

Navigate back to Table 1..

## Table 2. Long description

The table is organized into three main sections: social quantities, social qualities, and solitary qualities. For each variable, columns display B with 95 percent confidence interval, O R or beta with 95 percent confidence interval, and p value. In social quantities, ‘With any company’ shows B 0.01 (minus 0.01 to 0.02), O R 1.01 (0.99 to 1.02), p 0.48; ‘With familiar people’ B minus 0.01 (minus 0.04 to 0.02), O R 0.99 (0.96 to 1.02), p 0.60; ‘With less-familiar persons’ B minus 0.003 (minus 0.02 to 0.02), O R 1.00 (0.98 to 1.02), p 0.74. In social qualities, ‘Prefer to be alone’ B 0.04 (0.01 to 0.07), beta 0.05 (0.01 to 0.09), p 0.01 (significant); ‘Find company pleasant’ B minus 0.02 (minus 0.04 to minus 0.01), beta minus 0.06 (minus 0.11 to minus 0.02), p 0.008 (significant); ‘Feel safe in-company’ B minus 0.02 (minus 0.04 to minus 0.002), beta minus 0.05 (minus 0.10 to minus 0.01), p 0.03 (significant); ‘Feel judged’ B 0.01 (minus 0.01 to 0.04), beta 0.02 (minus 0.04 to 0.07), p 0.35; ‘Feel belonged’ B minus 0.02 (minus 0.03 to minus 0.001), beta minus 0.06 (minus 0.10 to minus 0.01), p 0.04 (significant). In solitary qualities, ‘Pleasant when alone’ B 0.01 (minus 0.02 to 0.03), beta 0.02 (minus 0.05 to 0.09), p 0.61; ‘Prefer company’ B minus 0.01 (minus 0.03 to 0.01), beta minus 0.04 (minus 0.11 to 0.03), p 0.25; ‘Feel safe when alone’ B minus 0.01 (minus 0.04 to 0.01), beta minus 0.04 (minus 0.11 to 0.02), p 0.29. Statistically significant associations (p less than 0.05) are found only in social qualities, specifically for ‘Prefer to be alone’, ‘Find company pleasant’, ‘Feel safe in-company’, and ‘Feel belonged’. All models are adjusted for age, sex, and total S C L dash 90. Abbreviations: A Q is Autism Spectrum Quotient, B is unstandardized regression coefficient, C I is confidence interval, O R is odds ratio, beta is standardized regression coefficient.

Navigate back to Table 2..

## Table 3. Long description

The table has five rows for social qualities: Prefer to be alone, Find company pleasant, Feel safe in-company, Feel judged, and Feel belonged. Each row has two sets of columns: with familiar people and with less-familiar people. For ‘Prefer to be alone’, B is 0.01 with 95 percent confidence interval negative 0.02 to 0.04 and p is 0.41 for familiar people; B is 0.02 with 0.001 to 0.04 and p is 0.04 for less-familiar people, which is significant. For ‘Find company pleasant’, B is negative 0.03 with negative 0.04 to negative 0.01 and p is 0.001 for familiar people, significant; B is negative 0.01 with negative 0.02 to 0.002 and p is 0.11 for less-familiar people. For ‘Feel safe in-company’, B is negative 0.03 with negative 0.05 to negative 0.01 and p is less than 0.001 for familiar people, significant; B is 0.01 with negative 0.003 to 0.02 and p is 0.18 for less-familiar people. For ‘Feel judged’, B is 0.02 with negative 0.01 to 0.04 and p is 0.18 for familiar people; B is 0.003 with negative 0.01 to 0.02 and p is 0.64 for less-familiar people. For ‘Feel belonged’, B is negative 0.03 with negative 0.05 to negative 0.02 and p is less than 0.001 for familiar people, significant; B is negative 0.01 with negative 0.01 to 0.003 and p is 0.21 for less-familiar people. All models are adjusted for age, sex, and total S C L dash 90. Significant p values are bolded. A Q stands for Autism Spectrum Quotient, B for unstandardized regression coefficient, and C I for confidence interval.

Navigate back to Table 3..

## Figure 1. Long description

Starting at the top left, panel A plots ‘Find company pleasant’ against total A Q scores from 0 to 50. The red line (without familiar people) starts high near 7 and decreases steadily, while the blue line (with familiar people) is lower and relatively flat, with a significant interaction p equals point zero zero one. Panel B, top center, shows ‘Feel safe in company’ with a similar pattern: the red line starts near 7.5 and decreases, the blue line is lower and flat, interaction p less than point zero zero one. Panel C, top right, ‘Feel belonged’, again shows the red line starting high and decreasing, blue line flat and lower, interaction p less than point zero zero one. Panel D, bottom left, ‘Prefer to be alone’, both lines start near zero, with the blue line (with familiar people) increasing more sharply than the red line, interaction p equals point four one. Panel E, bottom right, ‘Feel judged’, both lines are relatively flat, blue line slightly higher, interaction p equals point one eight. All panels include shaded regions representing ninety-five percent confidence intervals. The legend at bottom left identifies blue as with familiar people and red as without familiar people.

Navigate back to Figure 1..

## Figure 2. Long description

There are five panels labeled A through E, arranged left to right, top to bottom. Each panel plots predicted mean ratings with 95 percent confidence intervals for two groups: without less familiar people (blue) and with less familiar people (red), across total A Q scores from 0 to 50. Panel A, y-axis labeled ‘Find company pleasant’, shows both groups declining as A Q increases, with a steeper decline for the red group; interaction p equals point one one. Panel B, y-axis ‘Feel safe in company’, shows a similar pattern with both groups declining, red group lower; interaction p equals point one eight. Panel C, y-axis ‘Feel belonged’, shows both groups declining slightly, red group consistently lower; interaction p equals point two one. Panel D, y-axis ‘Prefer to be alone’, shows both groups increasing, red group higher and diverging more as A Q increases; interaction p equals point zero four. Panel E, y-axis ‘Feel judged’, shows both groups increasing slightly, red group consistently higher; interaction p equals point six four. Legend at bottom left: blue for without less familiar people, red for with less familiar people.

Navigate back to Figure 2..
