# Supplementary material for: Quality over quantity: association between autistic traits and daily-life social interactions in adolescents and young adults from the TwinssCan cohort
Source: CNS Spectr. 2026 Jun 1;31(1):e14. doi: 10.1017/S1092852926100947 (PMC13732628; doi:10.1017/S1092852926100947)
Supplement: Supplementary file 1 [file S1092852926100947sup001.docx]

**Supplementary Materials**

**Table S1.** Multilevel mixed-effects ordered logistic regression testing for the associations of autistic traits with the qualities of social or solitary experiences.

| **Social qualities** | **B (95% CI)** | **p** |
| --- | --- | --- |
| Prefer to be alone | 0.03 (0.01 to 0.05) | **0.01** |
| Find company pleasant | -0.03 (-0.05 to -0.005) | **0.01** |
| Feel safe in-company | -0.03 (-0.05 to -0.002) | **0.03** |
| Feel judged | 0.01 (-0.01 to 0.04) | 0.40 |
| Feel belonged | -0.02 (-0.04 to 0.00005) | 0.05 |
| **Solitary qualities** | **B (95% CI)** | **p** |
| Pleasant when alone | 0.01 (-0.02 to 0.04) | 0.47 |
| Prefer company | -0.02 (-0.04 to 0.01) | 0.30 |
| Feel safe when alone | -0.02 (-0.05 to 0.02) | 0.37 |

Note: All models were adjusted for age, sex, and total SCL-90. Statistical significance (p < 0.05) is presented in bold. B = unstandardized regression coefficient; CI = confidence interval; AQ = Autism-Spectrum Quotient.

**Table S2** Sensitivity analysis for associations of autistic traits with the quantities of social encounters and the qualities of social or solitary experiences, additionally adjusted for time of day, day type, and total number of beep responses.

| **Social quantities** | **B (95% CI)** | **OR (95% CI)** | **p** |
| --- | --- | --- | --- |
| With any company | 0.01 (-0.01 to 0.02) | 1.01 (0.99 to 1.02) | 0.50 |
| With familiar people | -0.02 (-0.05 to 0.02) | 0.98 (0.95 to 1.02) | 0.34 |
| With less-familiar persons | -0.001 (-0.02 to 0.02) | 1.00 (0.98 to 1.02) | 0.94 |
| **Social qualities** | **B (95% CI)** | **β (95% CI)** | **p** |
| Prefer to be alone | 0.04 (0.01 to 0.07) | 0.05 (0.01 to 0.09) | **0.02** |
| Find company pleasant | -0.02 (-0.04 to -0.01) | -0.07 (-0.11 to -0.02) | **0.006** |
| Feel safe in-company | -0.02 (-0.04 to -0.003) | -0.05 (-0.10 to -0.01) | **0.03** |
| Feel judged | 0.02 (-0.01 to 0.04) | 0.03 (-0.03 to 0.08) | 0.18 |
| Feel belonged | -0.02 (-0.04 to -0.003) | -0.06 (-0.11 to -0.02) | **0.02** |
| **Solitary qualities** | **B (95% CI)** | **β (95% CI)** | **P** |
| Pleasant when alone | 0.01 (-0.01 to 0.03) | 0.03 (-0.04 to 0.10) | 0.39 |
| Prefer company | -0.01 (-0.03 to 0.01) | -0.04 (-0.11 to 0.03) | 0.25 |
| Feel safe when alone | -0.01 (-0.03 to 0.01) | -0.03 (-0.10 to 0.04) | 0.39 |

Note: All models were adjusted for age, sex, and total SCL-90. Statistical significance (p *<* 0.05) is presented in bold. Standardized coefficients were calculated based on the total latent variance (the sum of all random-effect and residual variance components). B = unstandardized regression coefficient; CI = confidence interval; OR = odds ratio; β = standardized regression coefficient; AQ = Autism-Spectrum Quotient.

**Table S3** Associations between AQ subscale scores and the quality and quantity of social interactions.

| **Outcome** | **AQ subscale** | | | | | | | | | | | | | | |
| --- | --- | --- | --- | --- | --- | --- | --- | --- | --- | --- | --- | --- | --- | --- | --- |
|  | **Social skill** | | | **Attention switching** | | | **Attention to detail** | | | **Communication** | | | **Imagination** | | |
|  | **B (95% CI)** | **P** | **Q** | **B (95% CI)** | **P** | **q** | **B (95% CI)** | **P** | **q** | **B (95% CI)** | **p** | **Q** | **B (95% CI)** | **p** | **q** |
| **Social quantities** |  |  |  |  |  |  |  |  |  |  |  |  |  |  |  |
| **Being alone** | 0.02  (-0.03 to 0.06) | 0.53 | 1.0 | 0.01  (-0.04 to 0.06) | 0.66 | 1.0 | -0.02  (-0.07 to 0.02) | 0.27 | 1.0 | -0.01  (-0.06 to 0.04) | 0.71 | 1.0 | -0.04  (-0.10 to 0.01) | 0.11 | 1.0 |
| **With familiar persons** | -0.01  (-0.09 to 0.08) | 0.90 | 1.0 | 0.002  (-0.09 to 0.09) | 0.96 | 1.0 | -0.03  (-0.10 to 0.05) | 0.46 | 1.0 | -0.01  (-0.11 to 0.08) | 0.77 | 1.0 | -0.02  (-0.11 to 0.08) | 0.74 | 1.0 |
| **With less-familiar persons** | 0.001  (-0.05 to 0.05) | 0.98 | 1.0 | -0.02  (-0.07 to 0.04) | 0.50 | 1.0 | -0.01  (-0.05 to 0.04) | 0.80 | 1.0 | 0.03  (-0.02 to 0.09) | 0.26 | 1.0 | -0.03  (-0.09 to 0.02) | 0.24 | 1.0 |
| **Social qualities** |  |  |  |  |  |  |  |  |  |  |  |  |  |  |  |
| **Prefer to be alone** | 0.15  (0.06 to 0.23) | **0.001** | **0.01** | 0.08  (-0.02 to 0.17) | 0.10 | 0.58 | 0.04  (-0.04 to 0.12) | 0.36 | 1.0 | 0.06  (-0.04 to 0.15) | 0.25 | 0.96 | -0.003  (-0.10 to 0.09) | 0.95 | 1.0 |
| **Find company pleasant** | -0.08  (-0.12 to -0.03) | **0.001** | **0.01** | -0.06  (-0.11 to -0.01) | 0.01 | 0.06 | 0.02  (-0.02 to 0.06) | 0.40 | 0.93 | -0.02  (-0.07 to 0.03) | 0.41 | 0.93 | -0.05  (-0.1 to 0) | 0.050 | 0.05 |
| **Feel safe in-company** | -0.07  (-0.13 to -0.01) | 0.02 | 0.20 | -0.03  (-0.09 to 0.03) | 0.28 | 1.0 | -0.002  (-0.05 to 0.05) | 0.95 | 1.0 | -0.02  (-0.08 to 0.04) | 0.46 | 1.0 | -0.07  (-0.13 to 0) | 0.04 | 0.23 |
| **Feel judged** | 0.004  (-0.07 to 0.08) | 0.92 | 1.0 | 0.08  (-0.002 to 0.15) | 0.06 | .64 | 0.05  (-0.02 to 0.11) | 0.18 | 0.90 | 0.01  (-0.06 to 0.09) | 0.72 | 1.0 | -0.05  (-0.13 to 0.03) | 0.23 | 0.90 |
| **Feel belonged** | -0.06  (-0.10 to -0.01) | 0.01 | 0.17 | -0.04  (-0.09 to 0.01) | 0.10 | .52 | 0.004  (-0.04 to 0.05) | 0.86 | 1.0 | -0.01  (-0.06 to 0.04) | 0.59 | 1.0 | -0.04  (-0.09 to 0.01) | 0.14 | 0.52 |
| **Solitary qualities** |  |  |  |  |  |  |  |  |  |  |  |  |  |  |  |
| **Pleasant when alone** | 0.04  (-0.02 to 0.09) | 0.20 | 0.78 | 0.00  (-0.06 to 0.06) | 0.99 | 1.0 | 0.05  (0.002 to 0.11) | 0.04 | 0.46 | -0.02  (-0.08 to 0.04) | 0.55 | 1.0 | -0.05  (-0.12 to 0.01) | 0.09 | 0.50 |
| **Prefer company** | -0.07  (-0.13 to -0.01) | 0.02 | 0.25 | -0.02  (-0.09 to 0.04) | 0.45 | 1.0 | -0.02  (-0.08 to 0.03) | 0.36 | 1.0 | -0.004  (-0.07 to 0.06) | 0.91 | 1.0 | 0.03  (-0.03 to 0.10) | 0.33 | 1.0 |
| **Feel safe when alone** | -0.01  (-0.08 to 0.06) | 0.71 | 1.0 | -0.01  (-0.08 to 0.07) | 0.87 | 1.0 | 0.00  (-0.06 to 0.06) | 0.99 | 1.0 | -0.04  (-0.12 to 0.03) | 0.27 | 1.0 | -0.06  (-0.13 to 0.01) | 0.11 | 1.0 |

Note: All models were adjusted for age, gender, and total SCL-90. Statistical significance (Bonferroni-corrected p < 0.01 or q < 0.05) is presented in bold. B = unstandardized regression coefficient; CI = confidence interval; AQ = Autism-Spectrum Quotient.

**Table S4** Multilevel mixed-effects ordered logistic regression testing for the associations between AQ subscale scores and the quality of social interactions.

| **Outcome** | **AQ subscale** | | | | | | | | | | | | | | |
| --- | --- | --- | --- | --- | --- | --- | --- | --- | --- | --- | --- | --- | --- | --- | --- |
|  | **Social skill** | | | **Attention switching** | | | **Attention to detail** | | | **Communication** | | | **Imagination** | | |
|  | **B (95% CI)** | **P** | **q** | **B (95% CI)** | **p** | **q** | **B (95% CI)** | **p** | **q** | **B (95% CI)** | **p** | **q** | **B (95% CI)** | **p** | **q** |
| **Social qualities** |  |  |  |  |  |  |  |  |  |  |  |  |  |  |  |
| **Prefer to be alone** | 0.11  (0.05 to 0.17) | **<0.001** | **0.01** | 0.06  (-0.01 to 0.13) | 0.09 | 0.53 | 0.03  (-0.03 to 0.09) | 0.31 | 0.89 | 0.04  (-0.03 to 0.11) | 0.30 | 0.89 | 0.002  (-0.07 to 0.07) | 0.96 | 1.0 |
| **Find company pleasant** | -0.09  (-0.15 to -0.03) | **0.002** | **0.02** | -0.08  (-0.14 to -0.02) | 0.01 | 0.06 | 0.02  (-0.03 to 0.07) | 0.39 | 1.0 | -0.02  (-0.08 to 0.04) | 0.58 | 1.0 | -0.06  (-0.12 to 004) | 0.07 | 0.26 |
| **Feel safe in-company** | -0.09  (-0.16 to -0.02) | 0.012 | 0.13 | -0.04  (-0.12 to 0.03) | 0.23 | 0.89 | 0.003  (-0.06 to 0.07) | 0.91 | 1.0 | -0.03  (-0.10 to 0.05) | 0.50 | 1.0 | -0.08  (-0.15 to -0.001) | 0.05 | 0.26 |
| **Feel judged** | -0.003  (-0.07 to 0.07) | 0.94 | 1.0 | 0.07  (-0.001 to 0.15) | 0.05 | 0.60 | 0.04  (-0.02 to 0.11) | 0.22 | 0.89 | 0.01  (-0.06 to 0.09) | 0.73 | 1.0 | -0.05  (-0.13 to 0.03) | 0.23 | 0.89 |
| **Feel belonged** | -0.07  (-0.14 to -0.01) | 0.02 | 0.21 | -0.06  (-0.12 to 0.01) | 0.09 | 0.49 | 0.01  (-0.05 to 0.07) | 0.77 | 1.0 | -0.02  (-0.08 to 0.05) | 0.61 | 1.0 | -0.05  (-0.12 to 0.02) | 0.15 | 0.57 |
| **Solitary qualities** |  |  |  |  |  |  |  |  |  |  |  |  |  |  |  |
| **Pleasant when alone** | 0.07  (-0.02 to 0.15) | 0.11 | 0.43 | -0.001  (-0.10 to 0.08) | 0.86 | 1.0 | 0.08  (0.004 to 0.16) | 0.04 | 0.43 | -0.01  (-0.11 to 0.08) | 0.77 | 1.0 | -0.08  (-0.17 to 0.02) | 0.11 | 0.43 |
| **Prefer company** | -0.10  (-0.18 to -0.02) | 0.02 | 0.22 | -0.02  (-0.11 to 0.06) | 0.57 | 1.0 | -0.03  (-0.10 to 0.05) | 0.49 | 1.0 | -0.006  (-0.09 to 0.08) | 0.89 | 1.0 | 0.04  (-0.05 to 0.13) | 0.34 | 1.0 |
| **Feel safe when alone** | -0.01  (-0.11 to 0.09) | 0.80 | 1.0 | -0.01  (-0.12 to 0.10) | 0.85 | 1.0 | 0.004  (-0.09 to 0.09) | 0.93 | 1.0 | -0.06  (-0.17 to 0.05) | 0.29 | 1.0 | -0.08  (-0.18 to 0.03) | 0.17 | 1.0 |

Note: All models were adjusted for age, gender, and total SCL-90. Statistical significance (Bonferroni-corrected p < 0.01 or q < 0.05) is presented in bold. B = unstandardized regression coefficient; CI = confidence interval; AQ = Autism-Spectrum Quotient.

**Table S5** Multilevel mixed-effects ordered logistic regression testing for the interacting effects of being with familiar or less-familiar company and autistic traits on the quality of social interactions.

| **Social qualities** | **With familiar people** | | **With less-familiar people** | |
| --- | --- | --- | --- | --- |
|  | **B (95% CI)** | **p** | **B (95% CI)** | **p** |
| **Prefer to be alone** | 0.01 (-0.01 to 0.03) | 0.48 | 0.01 (0.001 to 0.03) | **0.04** |
| **Find company pleasant** | -0.03 (-0.05 to -0.01) | **0.003** | -0.01 (-0.02 to 0.003) | 0.13 |
| **Feel safe in-company** | -0.04 (-0.06 to -0.02) | **<0.001** | 0.01 (-0.01 to 0.02) | 0.31 |
| **Feel judged** | 0.01 (-0.01 to 0.03) | 0.28 | 0.01 (-0.01 to 0.02) | 0.37 |
| **Feel belonged** | -0.05 (-0.07 to -0.03) | **<0.001** | -0.01 (-0.02 to 0.01) | 0.39 |

Note: All models were adjusted for age, gender, and total SCL-90. Statistical significance (Bonferroni-corrected p < 0.05) is presented in bold. *B* = unstandardized regression coefficient; CI = confidence interval; AQ = Autism-Spectrum Quotient.

**Table S6** Sensitivity analysis for interacting effects of autistic traits and the presence of familiar or less-familiar people on social qualities, additionally adjusted for time of day, day type, and total number of beep responses.

| **Social qualities** | **With familiar people** | | **With less-familiar people** | | |
| --- | --- | --- | --- | --- | --- |
|  | **B (95% CI)** | **p** | **B** | **95% CI** | **p** |
| Prefer to be alone | 0.01 (-0.02 to 0.05) | 0.47 | 0.02 (0.004 to 0.04) | | **0.02** |
| Find company pleasant | -0.02 (-0.04 to -0.01) | **0.006** | -0.01 (-0.02 to 0.002) | | 0.10 |
| Feel safe in-company | -0.03 (-0.05 to -0.01) | **0.002** | 0.01 (-0.01 to 0.02) | | 0.29 |
| Feel judged | 0.02 (-0.01 to 0.04) | 0.21 | 0.001 (-0.01 to 0.01) | | 0.89 |
| Feel belonged | -0.03 (-0.05 to -0.01) | **<0.001** | -0.01 (-0.02 to 0.001) | | 0.07 |

Note: All models were adjusted for age, sex, and total SCL-90. Statistical significance (p < 0.05) is presented in bold. B = unstandardized regression coefficient; CI = confidence interval; AQ = Autism-Spectrum Quotient.

**Table S7** Interacting effects of being with familiar or less-familiar company and AQ Social Skill subscale on the quality of social interactions.

| **Social qualities** | **Multilevel tobit regression** | | | | | | **Multilevel mixed-effects ordered logistic regression** | | | | | |
| --- | --- | --- | --- | --- | --- | --- | --- | --- | --- | --- | --- | --- |
|  | **With familiar people** | | | **With less-familiar people** | | | **With familiar people** | | | **With less-familiar people** | | |
|  | **B (95% CI)** | **p** | **q** | **B (95% CI)** | **p** | **q** | **B (95% CI)** | **p** | **q** | **B (95% CI)** | **p** | **q** |
| **Prefer to be alone** | 0.02  (-0.08 to 0.12) | 0.72 | 1.0 | 0.04  (-0.02 to 0.09) | 0.17 | 0.67 | 0.004  (-0.06 to 0.07) | 0.92 | 1.0 | 0.03  (-0.01 to 0.07) | 0.17 | 0.65 |
| **Find company pleasant** | -0.08  (-0.13 to -0.03) | **0.002** | **0.02** | -0.03  (-0.06 to -0.002) | 0.04 | 0.22 | -0.09  (-0.16 to -0.03) | **0.005** | 0.05 | -0.04  (-0.07 to -0.003) | 0.03 | 0.20 |
| **Feel safe in-company** | -0.09  (-0.14 to -0.03) | **0.003** | **0.01** | 0.01  (-0.02 to 0.04) | 0.49 | 1.0 | -0.10  (-0.16 to -0.04) | **0.002** | **0.009** | 0.01  (-0.02 to 0.05) | 0.46 | 1.0 |
| **Feel judged** | 0.07  (0.002 to 0.14) | 0.04 | 0.44 | -0.003  (-0.04 to 0.04) | 0.87 | 1.0 | 0.07  (0.01 to 0.13) | 0.03 | 0.37 | 0.004  (-0.03 to 0.04) | 0.85 | 1.0 |
| **Feel belonged** | -0.08  (-0.03 to -0.04) | **0.001** | **0.003** | -0.02  (-0.05 to 0.004) | 0.09 | 0.33 | -0.14  (-0.20 to -0.07) | **<0.001** | **0.001** | -0.02  (-0.06 to 0.01) | 0.22 | 0.56 |

Note: All models were adjusted for age, gender, and total SCL-90. Statistical significance (Bonferroni-corrected p < 0.01 or q < 0.05) is presented in bold. B = unstandardized regression coefficient; CI = confidence interval; AQ = Autism-Spectrum Quotient.

**Rate from 1 (‘Not’) to 7 (‘very’)**

Who am I with?

With company (choose≥1) □ Partner □ Family living in the house □ Family living away □ Friends □ Colleagues □ Acquaintances □ Strangers/Others

I'd rather be alone.

I like this company.

In this company, I feel SAFE.

In this company, I feel JUDGED.

In this company, I belong.

Nobody

I like to be alone.

I'd like to be in the company of other people.

I feel SAFE.

**Figure S1.** The flowchart of ESM items used in the current analysis. Items assessing momentary experiences are shown in blue and were rated on a scale from 1 to 7, with higher scores indicating more agreement with the statement.

**Figure S2.** Marginal predictions of ratings for the quality of social experiences in the presence of solely familiar people, solely less-familiar people, or mixed, varying by AQ Total scores. Predicted means with 95% CI and p-values of the interaction between total AQ and social context, using less-familiar only as the reference, are presented. (A) Finding company pleasant, (B) Feeling safe in-company, (C) Feeling belonged, (D) Prefer to be alone, (E) Feeling judged.

**Figure S3.** Linear prediction of social qualities in the presence or absence of familiar people varying by AQ Social Skill subscale. Age, sex, and SCL-90 were kept constant at the mean values. The shaded area represents 95% CI of the prediction. P-values represent the statistical significance of an interaction between social skills and the presence of familiar people in predicting social qualities. (A) Prefer to be alone; (B) Finding company pleasant; (C) Feeling safe in-company; (D) Feeling judged; (E) Feeling belonged.

**Figure S4.** Linear prediction of social qualities in the presence or absence of less-familiar people varying by AQ Social Skill subscale. Age, sex, and SCL-90 were kept constant at the mean values. The shaded area represents 95% CI of the prediction. P-values represent the statistical significance of an interaction between social skills and the presence of less familiar people in predicting social qualities. (A) Prefer to be alone; (B) Finding company pleasant; (C) Feeling safe in-company; (D) Feeling judged; (E) Feeling belonged.
